# The need for obtaining accurate nationwide estimates of diabetes prevalence in India - Rationale for a national study on diabetes

**Published:** 2011-04

**Authors:** R.M. Anjana, M.K. Ali, R. Pradeepa, M. Deepa, M. Datta, R. Unnikrishnan, M. Rema, V. Mohan

**Affiliations:** *Madras Diabetes Research Foundation & Dr. Mohan’s Diabetes Specialities Centre, WHO Collaborating Centre for Noncommunicable Diseases, Prevention & Control, Chennai, India*; * *Hubert Department of Global Health, Rollins School of Public Health, Emory University, Atlanta, USA*

**Keywords:** Complications, diabetes, India, nationwide estimates, prevalence

## Abstract

According to the World Diabetes Atlas, India is projected to have around 51 million people with diabetes. However, these data are based on small sporadic studies done in some parts of the country. Even a few multi-centre studies that have been done, have several limitations. Also, marked heterogeneity between States limits the generalizability of results. Other studies done at various time periods also lack uniform methodology, do not take into consideration ethnic differences and have inadequate coverage. Thus, till date there has been no national study on the prevalence of diabetes which are truly representative of India as a whole. Moreover, the data on diabetes complications is even more scarce. Therefore, there is an urgent need for a large well-planned national study, which could provide reliable nationwide data, not only on prevalence of diabetes, but also on pre-diabetes, and the complications of diabetes in India. A study of this nature will have enormous public health impact and help policy makers to take action against diabetes in India.

The prevalence of diabetes mellitus is growing rapidly worldwide and is reaching epidemic proportions^1^,^2^. It is estimated that there are currently 285 million people with diabetes worldwide and this number is set to increase to 438 million by the year 2030^3^. The major proportion of this increase will occur in developing countries of the world where the disorder predominantly affects younger adults in the economically productive age group^4^. There is also consensus that the South Asia region will include three of the top ten countries in the world (India, Pakistan and Bangladesh) in terms of the estimated absolute numbers of people with diabetes^3^.

Although the exact reasons why Asian Indians are more prone to type 2 diabetes at a younger age and premature cardiovascular disease (CVD) remain speculative, there is a growing body of evidence to support the concept of the “Asian Indian Phenotype”^5^. This term refers to the peculiar metabolic features of Asian Indians characterized by a propensity to excess visceral adiposity, dyslipidaemia with low HDL cholesterol, elevated serum triglycerides and increased small, dense LDL cholesterol, and an increased ethnic (possibly genetic) susceptibility to diabetes and premature coronary artery disease^5^,^6^.

However, to view it in the proper perspective, the estimates regarding the number of people with diabetes in India are derived from a few scattered studies conducted in different parts of the country. There have been a few multi-centre studies such as the ICMR studies conducted in 1979^7^ and 1991^8^, National Urban Diabetes Survey (NUDS) in 2001^9^, the Prevalence of Diabetes in India Study (PODIS) in 2004^10^ and the WHO-ICMR NCD Risk factor Surveillance study in 2008^11^. However, to date, there has been no national study which has looked at the prevalence of diabetes in India as a whole, covering all the States of the country or indeed, even in any single s0 tate with comprehensive urban and rural representation. In this article we review the published studies on the prevalence of diabetes and its complications in India and make a case for the need for a truly representative national study on the prevalence of diabetes in India.

## The rise of non communicable diseases in India

**Fig. 1:**
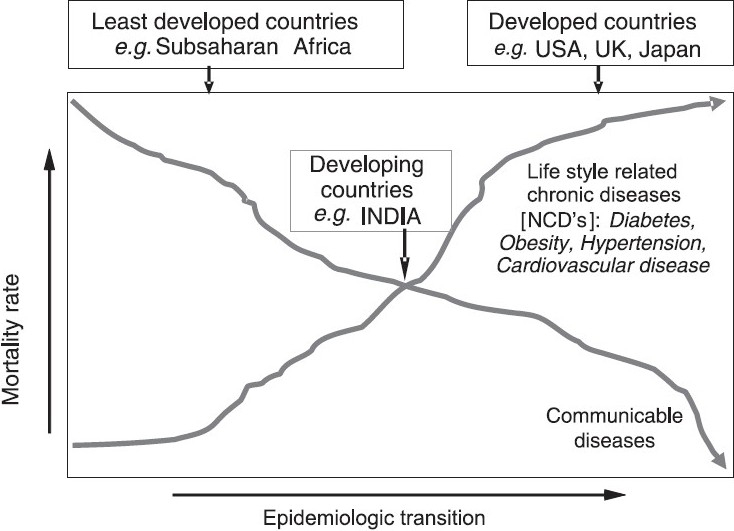
Epidemiologic transition of communicable vs non-communicable diseases.

In countries like the United States, Germany, the United Kingdom and Japan, the prevalence of communicable diseases is much lower compared to chronic non-communicable diseases (NCD). In India, as in other low and middle income countries, diabetes and other NCDs are relatively overshadowed by the continued burden of communicable and nutrition-related diseases. While these health threats are still present (albeit, slowly decreasing), the rise of NCDs has been rather rapid. According to the World Health Report 2005^12^, NCDs already contribute to 52 per cent of the total mortality in India and these figures are expected to increase to 69 per cent by the year 2030^13^. Therefore, countries like India are currently facing an epidemiologic transition with a ’double burden’ of disease as shown in Fig. 1.

Globally, many of the risk factors for NCDs are lifestyle related and can be prevented. Ebrahim & Smeeth *et al*^14^ conclude that NCDs in low and middle income countries are a priority and that it would be a serious mistake to ignore their prevention and control. Another study^15^ which looked at the burden of NCDs in South Asia reports that ‘research and surveillance is urgently needed with new studies following more rigorous and standardized methods to assess the true extent and impact of NCDs in South Asia’.

The World Health Organization is urging health decision makers to develop effective prevention strategies to halt the rising trend of NCDs through the control of risk factors. Although most of the developed world has reacted by instituting pragmatic measures for risk factor control, the global burden of NCDs continues to grow. This is largely because developing countries like India provide the bulk of numbers of individuals with diabetes and other NCDs and in most developing countries the focus is still on infectious diseases and NCDs continue to be neglected. Thus, there is an urgent need for strategies to detect and control diabetes and other NCDs in developing countries.

## Epidemiological studies in India

Ancient Indian texts make mention of the disease “*Madhumeha*” which would correspond to the modern term “Diabetes mellitus”, suggesting that diabetes must have been present in India even before 2500 BC. Although, there is no evidence as to how prevalent the condition was, a recent article hypothesizes that it could have been quite common in India, even in ancient times^16^.

Tables I^17^–^66^ and II^7^–^11^,^67^ list the published studies on the prevalence of diabetes in India till date. As shown in Table II, there are only six studies which have sampled respondents at multiple locations. The ICMR survey done in the 1970s studied urban and rural areas but was limited to six regions^7^. Given the major socio-demographic and economic changes as well as technological advances in the past 30 years, most of this data are outdated and not applicable to India’s current population. The National Urban Diabetes Survey (NUDS) investigated prevalence of diabetes in 6 large metropolitan cities (“metros”) of India in 2001, but there was no rural component^9^. The Prevalence of Diabetes in India Study (PODIS) included smaller towns and villages but excluded the metros and big cities^10^,^68^. The WHO-ICMR NCD Risk Factor Surveillance Study described the self-reported prevalence of diabetes in 6 centers, but no objective blood sugar testing was done^11^.

**Table I:** A compilation of epidemiology studies on diabetes in different regions of India

Region		Urban				Rural			
Author, Place	Year of publication	n	Age (yr)	Method adopted for diagnosis	Prevalence (%)	n	Age (yr)	Method adopted for diagnosis	Prevalence (%)
*Northern region*:										
Berry *et al*, Chandigarh^17^	1966	3846	30+	US	2.9	-	-	-	-
Gour, Varanasi^18^	1966	2572	10+	US	2.7	-	-	-	-
Datta *et al*, Lucknow^19^	1973	2190	20+	RBG	1.1	-	-	-	-
Ahuja *et al*, Delhi^20^	1974	2783	15+	RBG	2.3	-	-	-	-
Varma, Delhi^21^	1974	2291	20+	RBG	2.7	-	-	-	-
Varma *et al*, Delhi^22^	1986	6878	20+	K	3.1	-	-	-	-
Tiwari & Bissaraya, Rewa^23^	1988	-	-	-	-	15000	-	RBG	1.9
Wander *et al*, Punjab^24^	1994	-	-	-	-	1100	30+	K + PG	4.6
Zargar *et al*, Srinagar^25^	2000	1538	40+	K + F+ PG*	5.2	4045	40+	-	4.0
Misra *et al*, Delhi^26^	2001	532	18+	K + F	10.3	-	-	-	-
Gupta *et al*, Jaipur^27^	2003	1091	20+	K + F	12.3	-	-	-	-
Gupta *et al*, Jaipur^28^	2004	458	20+	K + F	16.8	-	-	-	-
Agrawal *et al*, Rajasthan^29^	2004	-	-	-	-	782	20+	-	1.8
Prabhakaran *et al*, Delhi^30^	2005	2122	20-59	K+ F+ PG	15.0	-	-	-	-
Gupta *et al*, Jaipur^31^	2007	1127	20+	K + F	20.1	-	-	-	-
Kokiwar *et al*, Nagpur^32^	2007	-	-	-	-	924	30+	K+ F+ PG	3.7
Agrawal *et al*, Rajasthan^33^	2007	-	-	-	-	2099	20+	-	1.7
*Southern region*:									
Rao *et al*, Hyderabad^34^	1966	21396	20+	US	4.1	-	-	-	-
Viswanathan *et al*, Chennai^35^	1966	5030	20+	US	5.6	-	-	-	-
Datta *et al*, Pondicherry^36^	1966	2694	20+	US	0.7	-	-	-	-
Rao *et al*, Hyderabad^37^	1972	-	-	-	-	2006	20+	US	2.4
Vigg *et al*, Hyderabad^38^	1972	-	-	-	-	847	10+	RBG	2.5
Parameswara, Bangalore^39^	1973	25273	5+	RBG	2.3	-	-	-	-
Murthy *et al*, Tenali^40^	1984	-	-	-	-	848	15+	RBG	4.7
Ramachandran *et al*, Kudremukh^41^	1988	678	20+	K+ F+ PG	5.0	-	-	-	-
Ramaiya *et al*, Gangavati^42^	1990	-	-	-	-	765	30+	K+ F + PG	2.2
Ramachandran *et al*, Chennai^43^	1992	900	20+	K+ F+ PG*	8.2			-	
Ramachandran *et al*, Sriperumbudur^43^	1992	-	-	-	-	1038	20+	K + F+ PG*	2.4
Patandin *et al*, North Arcot^44^	1994	-	-	-	-	467	40+	K + PG*	4.9
Ramachandran *et al*, Chennai^45^	1997	2183	20+	K+ F+ PG	11.6	-	-	-	-
Bai *et al*, Chennai^46^	1999	1198	NA	K+ F+ PG	7.6	-	-	-	-
Kutty *et al*, Trivandrum^47^	2000	518	20+	RBG*	12.4	-	-	-	-
Joseph *et al*, Trivandrum^48^	2000	206	19+	K+ PG	16.3	-	-	-	-
Asha Bai *et al*, Chennai^49^	2000	26066	20+	K	2.9	-	-	-	-
Mohan *et al*, Chennai^50^	2001	1262	20+	K+ F+ PG	12.0	-	-	-	-
Mohan *et al*, Chennai^51^	2006	2350	20+	K+ F+ PG	15.5	-	-	-	-
Chow *et al*, Godavari^52^	2006	-	-	-	-	4535	30+	F*	13.2
Menon *et al*, Kochi^53^	2006	3069	18-80	K+ PG*	19.5	-	-	-	-
Ramachandran *et al*, Chennai^54^	2008	2192	20+	K+ F+ PG	18.6	-	-	-	-
*Eastern region*:									
Tripathy *et al*, Orissa^55^	1971	-	-	-	-	2447	10+	RBG	1.2
Chhetri *et al*, Kolkata^56^	1975	4000	20+	RBG	2.3	-	-	-	-
Shah *et al*, Guwahati^57^	1998	1016	20+	K+ PG	8.2	-	-	-	-
Singh *et al*, Manipur^58^	2001	1664	15+	K+ PG	4.0	-	-	-	-
Kumar *et al*, Kolkata^59^	2008	2160	20+	K+ F*	11.5	-	-	-	-
*Western region*:									
Patel *et al*, Mumbai^60^	1963	18243	20+	US	1.5	-	-	-	-
KEM Hospital, Mumbai^61^	1966	3200	20+	RBG	2.1	-	-	-	-
Gupta *et al*, Ahmedabad^62^	1978	3516	15+	RBG	3.0	-	-	-	-
Patel, Bhadlan^63^	1986	-	-	-	-	3374	10+	RBG	3.8
Iyer *et al*, Bardoli^64^	1987	-	-	-	-	1348	All	RBG	4.4
Iyer *et al*, Mumbai^65^	2001	520	20+	K+ F+ PG	7.5	-	-	-	-
Deo *et al*, Sindhudurg^66^	2006	-	-	-	-	1022	20+	K+ F+ PG	9.3

US, Urine sugar; RBG, random blood glucose; K, known diabetes; F, fasting blood glucose; PG, post glucose load

* Capillary blood glucose method

**Table II:** Multicentric studies on diabetes prevalence in India

Region			Urban				Rural			
Author	Place	Year of publication	n	Age (yr)	Method adopted for diagnosis	Prevalence (%)	n	Age (yr)	Method adopted for diagnosis	Prevalence (%)
Ahuja^7^ (Urban + Rural)	Ahmedabad		3496			3.7	3483			1.9
	Kolkata		3488			1.8	3515			1.5
	Cuttack	1979	3849	15+	K + PG*	2.0	2993	15+	K + PG*	1.6
	Delhi		2358			0.9	2308			1.5
	Pune		2796			1.9	2818			1.1
	Trivandrum		3090			1.8	-			-
Ahuja^8^ (Urban + Rural)	Delhi		2572			4.1	992			1.5
	Kalpa						999			0.4
	Trivandrum	1991		20+	K + PG*		1488	20+	K + PG*	1.3
	Kolkata						2375			0.8
	Ahmedabad						1294			3.9
Ramachandran *et al*^9^ (only Metros)	Delhi		2300		K + F+ PG*	11.6	-	-	-	-
	Bangalore		1359			12.4	-	-	-	-
	Chennai	2001	1668	20+	K+ PG*	13.5	-	-	-	-
	Hyderabad		1427			16.6	-	-	-	-
	Kolkata		2378			11.7	-	-	-	-
	Mumbai		2084		K +F+ PG*	9.3	-	-	-	-
Sadikot *et al*^10^ (Metros excluded)	National	2004	10617	25+	K +F+ PG*	5.9	7746	25+	K +F+ PG*	2.7
Ajay *et al*^67^ (Industrial cohort)	Delhi		3358			10.9	-	-	-	-
	Hyderabad		908			14.1	-	-	-	-
	Chennai	2008	492	20+	K +F+ PG*	10.4	-	-	-	-
	Bangalore		702			10.7	-	-	-	-
	Trivandrum		1098			16.6	-	-	-	-
Mohan *et al*^11^ (Urban + Rural)	Ballabgarh					4.8				1.1
	Chennai	2008	15230	15 - 64	K	8.7	13522	15 - 64	K	3.9
	Delhi					10.3				-
	Dibrugarh					5.5				0.6
	Nagpur					3.2				0.6
	Trivandrum					11.2				9.6

US, Urine sugar; RBG, random blood glucose; K, known diabetes; F, fasting blood glucose; PG, post glucose load

* Capillary blood glucose method

Scarcity of good quality epidemiological data is a serious limitation in developing countries like India. So far, the major source of population level estimates of diabetes in India has been *ad hoc* surveys in limited geographical regions. Table III gives the various limitations of existing studies of diabetes prevalence in India. Starting from the early 1960s, there have been over 60 studies (Table I & Table II) which have reported on the prevalence of diabetes in India. These studies are characterized by several limitations: regional, with small sample sizes, low response rates, use varied diagnostic criteria and sample designs, lack standardization, leading to measurement errors and incomplete reporting of results. To date, surveys have not managed to capture standardized measures of diet and physical activity, health service utilization, health care costs and the level of glycaemic control. In addition, a disproportionately large number of studies have examined the prevalence of diabetes in urban settings, to the exclusion of the rural population, where over 70 per cent of India’s population resides.

**Table III:** Limitations of existing studies of diabetes prevalence in India

(1)	Ad hoc surveys
(2)	Regional focus
(3)	Lack of uniform methodology
(4)	Small sample sizes
(5)	Rural representation inadequate
(6)	Incomplete diagnostic work
(7)	Use of varied diagnostic criteria
(8)	Use of varied sample designs
(9)	Inadequate coverages
(10)	Lack of standardization
(11)	Measurement errors
(12)	Done in different time periods

Thus, as is evident, there is not a single study which has looked at all the States and regions of India and none that has included urban and rural areas in addition to metropolitan cities. Indeed, as noted earlier, there is no study which looked at the prevalence of diabetes even in a representative sample of a single State of the country.

## Diabetes-related complications

Till the early 1990s, there were no population-based data on diabetes-related complications. Such data are of great significance since these represent the burden of the disease. Clinic-based data are subject to referral bias and only represent the profile of patients seen in that particular clinic. Table IV presents the studies on the prevalence of diabetes-related complications in India^69^–^92^. These studies have reported interesting differences in the patterns of complications seen in Asian Indians. For example, the prevalence of retinopathy^73^, nephropathy^80^, and peripheral vascular disease, appear to be lower^92^, while that of neuropathy appears to be similar to prevalence rates reported in the West^84^. The prevalence of cardiovascular disease on the other hand was shown to be higher^90^ than that reported in the West.

**Table IV:** Population and clinical based studies on prevalence of diabetes complications in India

Author	Year	Clinic/population based study	City/State	Prevalence (%)
*Retinopathy*:				
Rema *et al*^69^	1996	Clinic	Chennai	34.1
Ramachandran *et al*^70^	1999	Clinic	Chennai	23.7
Dandona *et al*^71^	1999	Population	Hyderabad	22.6
Narendran *et al*^72^	2002	Population	Palakkad	26.8
Rema *et al*^73^	2005	Population	Chennai	17.6
*Nephropathy*:				
John *et al*^74^	1991	Clinic	Vellore	Microalbuminuria: 19.7
				Diabetic nephropathy: 8.9
Gupta *et al*^75^	1991	Clinic	New Delhi	Microalbuminuria: 26.6
Yajnik *et al*^76^	1992	Clinic	Pune	Microalbuminuria: 23.0
Vijay *et al*^77^	1994	Clinic	Chennai	Proteinuria: 18.7
Mohan *et al*^78^	2000	Clinic	Chennai	Macroproteinuria with retinopathy: 6.9
Varghese *et al*^79^	2001	Clinic	Chennai	Microalbuminuria: 36.3
Unnikrishnan *et al*^80^	2006	Population	Chennai	Microalbuminuria : 26.9
				Overt nephropathy with diabetic retinopathy : 2.2
*Neuropathy*:				
Ramachandran *et al*^70^	1999	Clinic	Chennai	27.5
Ashok *et al*^81^	2002	Clinic	Chennai	19.1
Viswanathan V *et al*^82^	2005	Clinic	Chennai	17
Viswanathan V *et al*^82^	2005	Clinic	Vellore	16
Viswanathan V *et al*^82^	2005	Clinic	Delhi	9
Viswanathan V *et al*^82^	2005	Clinic	Madurai	14
Chanda *et al*^83^	2006	Clinic	Bangalore	64.1
Pradeepa *et al*^84^	2008	Population	Chennai	26.1
*Coronary artery disease*:				
Chaddha *et al*^85^	1990	Population	New Delhi	9.7
Raman Kutty *et al*^86^	1993	Population	Kerala	7.4
Mohan *et al*^87^	1995	Clinic	Chennai	17.8
Gupta *et al*^88^	1995	Population	Uttar Pradesh	7.9
Ramachandran *et al*^89^	1998	Population	Chennai	14.3
Ramachandran *et al*^70^	1999	Clinic	Chennai	11.4
Mohan *et al*^90^	2001	Population	Chennai	21.4
Gupta *et al*^91^	2002	Population	Rajasthan	8.2
*Peripheral vascular disease*:				
Premalatha *et al*^92^	2000	Population	Chennai	6.3

Diabetes is traditionally known as a “silent disease,” exhibiting no symptoms until it progresses to severe target organ damage^93^. Case detection, therefore, requires active and opportunistic screening efforts^94^. However, even where diagnosed, inadequate glycaemic control^95^–^97^ results in seriously disabling or life-threatening complications. As a result, diabetes is the leading cause of adult-onset blindness and kidney failure worldwide and is responsible for approximately 6 per cent of total global mortality, accounting for 3.8 million deaths in 2007^98^,^99^. Although South Asia currently has the highest number of diabetes-related deaths, accurate prevalence estimates of complications in large segments of the population are glaringly absent.

## Rationale for a national diabetes survey

India is a vast, heterogeneous country with an approximate population of 1.1 billion people, a complex socio-political history, immense diversity of culture, dialects and customs, public and privately-funded health infrastructure, and competing demands on human and structural resources. These factors together negate a single policy solution for the whole country and this underscores the importance of generating a robust, representative base of evidence that documents burdens of disease, identifies vulnerable populations and draws attention to disease determinants^100^,^101^. Approximately 742 million people in India live in rural areas^102^,^103^ where awareness of chronic diseases is extremely low^104^ and the ratio of unknown-to-known diabetes is 3:1 (compared to 1:1 in urban areas)^11^. Crude estimates suggest that type 2 diabetes prevalence in rural areas is much lower (approximately 25-50%) than in urban areas^105^,^106^, although trend data are now suggesting that diabetes prevalence in rural areas is rapidly catching up with the urban estimates. In addition, given that the overwhelming majority of India’s population lives in rural areas and that there is a higher ratio of undiagnosed cases, the burden of diabetes and NCDs may be much greater in rural areas. Also, large disparities in human and infrastructural resource allocation between rural and urban areas are directly related to divergence in disease outcomes^107^,^108^. Therefore, the Government of India’s National Rural Health Mission will benefit greatly from more precise estimates of diabetes and NCD burden in all States of India. The gist of the rationale for a national diabetes survey in India is given in Table V.

**Table V:** Rationale for a national diabetes study

(1)	Rapid rise in the prevalence of diabetes in India.
(2)	Younger age of onset of diabetes in India leading to great economic and social burden.
(3)	Existing studies have limitations.
(4)	No study which is representative of even a whole State and thus no representative national figures.
(5)	Marked heterogeneity between States which limits the generalisability of results of small regional studies.
(6)	Multi-centre studies are also limited to either metros or small towns and villages and do not take into account all the geographical divisions.
(7)	Population based work on diabetes complications is sparse with no single study looking at all the complications in different regions of India.
(8)	To estimate the current burden of diabetes (as a model of NCDs) and its complications in India.
(9)	Need for such data to plan and develop national health policies.

## Significance and impact of a large representative national study

Given that there is a growing epidemic of diabetes in India^109^, reliable and informative epidemiological evidence is vital to quantify impacts and predictors of disease and facilitate formulation of prevention and control strategies. Effective prevention and care models have the potential to lower rates of target organ damage, disability and premature mortality, resulting in long term savings in health expenditure^110^,^111^. Currently, there are large data deficits regarding the distribution, trends, determinants and disease outcomes and where information is available, vast State-wise heterogeneity and variable quality limit its value.

A national study on diabetes called as the ICMR-INDIA DIABETES (ICMR-INDIAB) study is being planned which will address the following questions (*i*) What is the prevalence of diabetes in India?, (*ii*) What is the urban prevalence and what is the rural prevalence?, (*iii*) Are there really regional disparities in the prevalence of diabetes in India? and (*iv*) If so, are these differences due to differing dietary patterns (rice vs. wheat as staple food), or differences in levels of physical activity, or are there true ethnic differences in the susceptibility to diabetes even *within* the Asian Indian population? These are just some of the questions that will be answered by this large national study on diabetes.

**Fig. 2:**
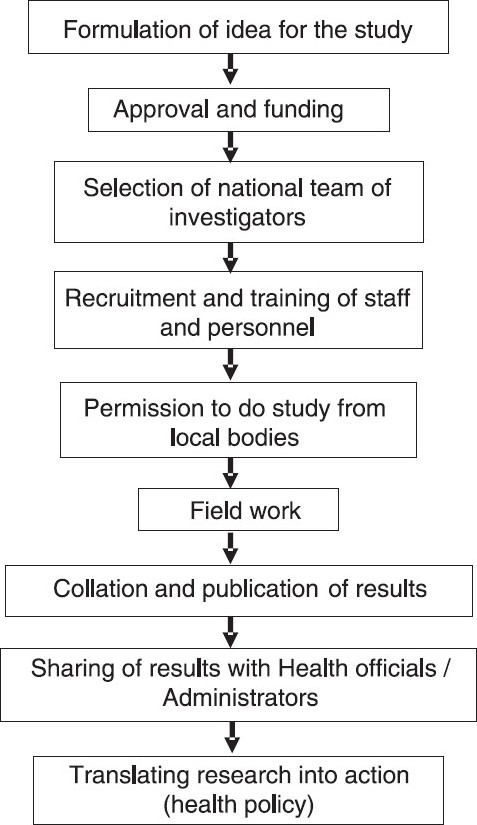
Flow chart to depict the study path.

A well-planned national study on diabetes like the ICMR-INDIAB study could provide a truly representative picture of diabetes in the whole nation. Such a study would provide reliable nationwide data, not only on prevalence of diabetes, but also on pre-diabetes and the metabolic syndrome. It can also be used to generate appropriate thresholds for serum lipid parameters for the country’s population. It could provide information on dietary patterns and physical activity for India as a whole, in addition to studying the genetic diversity of India in relation to NCDs in general, and diabetes in particular. This kind of data will be extremely informative and contribute to national and State level policy decision making. An additional component of the study would be to provide accurate data on all diabetes complications and this would once again be the first of its kind in the country. Even in rural areas, where literacy rates are low, the study would provide information about health and disease. In addition, training young investigators and personnel from the local areas could empower them with knowledge and technical skills which can be used for the betterment of the community as a whole. Further, enduring analyses and sub-analyses from a study of this magnitude will fuel the evolution of more research questions, including the potential to repeat measures to examine future trends. Fig. 2 presents a flow chart depicting the study pathway.

The challenges involved in doing a large national study are many - geographic barriers, social barriers, language barriers, cultural barriers and ethnic barriers are just to name a few. However, the major challenge will be to maintain the highest standards of quality to produce world class data.

In conclusion, despite recent advances in knowledge, the prevention and control of non communicable diseases like diabetes and CVD remain a major challenge in India^112^,^113^. Several important questions regarding the regional distribution, determinants, and interventions for diabetes remain unanswered. Thus the need for a large multi-State representative population-based study on the prevalence of diabetes and its complications and related metabolic NCDs like hypertension, obesity, dyslipidaemia and cardiovascular disease in India cannot be emphasized.
